# Time-lapse contact microscopy of cell cultures based on non-coherent illumination

**DOI:** 10.1038/srep14532

**Published:** 2015-10-13

**Authors:** Marion Gabriel, Dorothée Balle, Stéphanie Bigault, Cyrille Pornin, Stéphane Gétin, François Perraut, Marc R. Block, François Chatelain, Nathalie Picollet-D’hahan, Xavier Gidrol, Vincent Haguet

**Affiliations:** 1 CEA, iRTSV-BGE, F-38000 Grenoble, France; 2 INSERM, BGE, F-38000 Grenoble, France; 3 Université Grenoble Alpes, iRTSV-BGE, F-38000 Grenoble, France; 4 CEA, Léti-DOPT, F-38000 Grenoble, France; 5 CEA, Léti-DTBS, F-38000 Grenoble, France; 6 IAB, CRI INSERM/UJF U823, 38706 La Tronche, France; 7 CEA, IBS, F-38000 Grenoble, France

## Abstract

Video microscopy offers outstanding capabilities to investigate the dynamics of biological and pathological mechanisms in optimal culture conditions. Contact imaging is one of the simplest imaging architectures to digitally record images of cells due to the absence of any objective between the sample and the image sensor. However, in the framework of in-line holography, other optical components, *e.g.*, an optical filter or a pinhole, are placed underneath the light source in order to illuminate the cells with a coherent or quasi-coherent incident light. In this study, we demonstrate that contact imaging with an incident light of both limited temporal and spatial coherences can be achieved with sufficiently high quality for most applications in cell biology, including monitoring of cell sedimentation, rolling, adhesion, spreading, proliferation, motility, death and detachment. Patterns of cells were recorded at various distances between 0 and 1000 μm from the pixel array of the image sensors. Cells in suspension, just deposited or at mitosis focalise light into photonic nanojets which can be visualised by contact imaging. Light refraction by cells significantly varies during the adhesion process, the cell cycle and among the cell population in connection with every modification in the tridimensional morphology of a cell.

Cellular mechanisms involved in biological or pathological functions of living organisms are massively investigated using techniques of cell imagery. To facilitate handling of samples, standard processes of cell observation and characterisation rely on cell fixation. However, fixed-cell assays are an invasive technique which only permits observation of cells at endpoints of the experiments, with limited possibilities to extrapolate kinetics or reveal slow, transient or rare cellular events.

There is currently a growing need of time-lapse investigation for better characterisation of cell populations, *e.g.*, to study cell proliferation, morphology evolution, long-period cytotoxicity, cell variability, cell-cell interaction, cell-substrate interaction, motility or chemotaxis. This trend is supported by the strong desire, particularly in cancer and infectious disease research, to move to live-cell applications which are considered by many biologists as being far more biologically relevant than fixed-cell assays^1^. Despite the interest of collecting more history dependent information, live-cell assays are estimated to represent less than 10% of the total assays performed^2^.

Video microscopy instruments allow capturing images of cell cultures within cell friendly environments^3^,^4^,^5^. However, video microscopes provide less accurate regulation of temperature and CO_2_ ratio compared to cell culture incubators because of temperature inhomogeneity and unavoidable leaks through apertures in the enclosures. Video microscopes may also suffer from focus drift resulting from temperature gradient and mechanical vibrations.

Video microscopes and bench-top microscopes have very limited portability. The common approach in a laboratory is to bring the samples to be visualised toward the microscope rather than the opposite. Lack of compactness and portability of microscopy equipment is also noticeable in the field of lab-on-chip engineering where 2 cm-sized microfluidic devices have to be installed under a 50 cm-sized bench-top microscope for visualisation and manipulation of the devices. We believe that a better approach for observing tissue cultures and microfluidic systems would be to miniaturise the microscope so that it can provide *in situ* observations in a regulated CO_2_ incubator or on the collecting site. The resulting observations and measurements onto the cells would be far more physiological whether the cells were continuously cultured in their optimal temperature and pH conditions and were avoided mechanical stress during transport. The continuous supply of appropriate culture conditions is especially critical for cells requiring a particular environment, *e.g.*, low O_2_ conditions. Furthermore, limiting the environmental changes for the cells decreases the risk of bacterial or fungi contamination.

Recently, small-size microscopes have been achieved for use in life sciences applications. A fluorescence illumination system and a compact objective^6^ or, in a simpler configuration, a millimetre-sized lens^7^,^8^ were assembled in front of the camera module of a mobile phone for diagnostic imaging and telemedicine. Further miniaturisation of the entire imaging device was also carried out using minute optical components to build a centimetre-sized microscope^9^,^10^ as well as millimeter-sized^11^ and capsule^12^,^13^ endoscopes.

An alternative approach to reducing the dimensions of optics consists in avoiding any intermediary lens between the sample and the image sensor by placing the sample directly in front of the image sensor. The first works using this technique were the photograms of plants laid on a photosensitive paper^14^,^15^. Contact imaging, lensfree imaging and lensless imaging are similar terms currently in use in the literature to name this approach. Direct coupling of the sample and the image sensor has been applied for the two latter decades to biotechnology applications using digital imaging devices instead of photosensitive films. A single photodiode or a planar array of photodiodes have been employed for the reading of DNA chips^16^,^17^,^18^, quantification of molecular concentrations by absorbance^19^,^20^ and fluorescence^19^,^21^ spectrometry, routine inspection of protein crystals^22^, as well as detection and counting of stained or wetting film-covered viruses^23^, spores^24^, bacteria^25^,^26^ and cells^27^,^28^. Fluorescent imaging of labelled cells has also been achieved with an absorbance filter placed on the imaging area^29^, with the possible use of total internal reflection to increase excitation rejection and a light guide to limit fluorescence spreading^30^.

Recently, brightfield contact imaging of human cells has been made possible because of the dramatic reduction of the pixel size of image sensors which is now far smaller than the human cell dimensions (10-30 μm range)^31^,^32^,^33^,^34^,^35^,^36^,^37^,^38^,^39^. Lensfree imaging was applied to record contractions of cardiomyocyte colonies^40^, quantify motility of sperm cells^41^,^42^, visualise microvessels formed by endothelial cells^43^,^44^, classify prostate organoids grown in Matrigel^45^ and discriminate fast flowing cells^46^. Furthermore, we recently arrayed 96 image sensors on a printed circuit board to achieve time-lapse screening of cell cultures in a 96-well microtiter plate^22^,^47^,^48^.

Brightfield contact imaging of cells can be described by the theory of in-line holography^49^,^50^: the light diffracted by the cells (object beam) and the non-scattered light from the source (reference beam) interfere to form diffraction patterns, called holographic patterns or holograms, which are recorded by the image sensor. However, since most individual cells are transparent objects, the generated holograms are also partly deformed by the cells as the object beam is refracted when passing through the cells. In this article, the contribution of refraction by cells to the image collected by the image sensor is investigated. We show that the hologram of a cell is significantly shaped by refraction during the cell cycle as a result of considerable changes in the cell morphology.

In addition, within the framework of in-line holography, the biological samples were preferably illuminated with a coherent or quasi-coherent incident light in most previous studies in order to generate non-blurred interference patterns^23^,^26^,^31^,^32^,^38^,^39^,^41^,^42^,^43^,^44^,^45^. A monochromatic, spatially coherent light has been achieved by a laser beam focused through a pinhole by a microscope objective^31^,^32^. A quasi-coherent incident light is typically produced by a point source made by a pinhole^26^,^32^,^39^,^41^,^44^,^45^ or an optical fibre^23^,^42^,^43^, back-illuminated by a colour LED with a low bandwidth (*e.g.*, <20 nm). Fewer works have explored the ability to capture images of non-labelled biological samples using partially non-coherent incident light^37^,^40^. In this article, we demonstrate that contact imaging of cells can be achieved with sufficiently high quality for a wide range of cell biology applications even when using a light source of both limited temporal and spatial coherences and in the absence of any temporal and spatial filters between the light source and the sample.

## Results

### Contact imaging architecture

**Figure 1:**
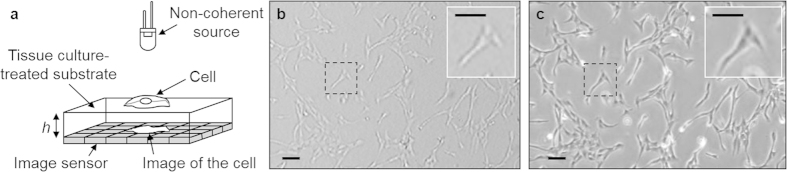
Contact imaging of cells. (**a**) Principle of the cell imaging device. The image of the cell population placed at a height *h* above the pixel array of the image sensor is projected onto it by a light source of limited temporal and spatial coherences. (**b**) Image of RPE1 cells adhered on a fibronectin-coated glass slide (h = 175 μm). (**c**) Same area, visualised by phase contrast microscopy at magnification 4×, as in (**b**). The number, position and shape of the cells are identical in (**b**) and (**c**), demonstrating the reliability of the contact imaging device. Scale bars: 100 μm.

To investigate the effect of refraction by cells on the formed holograms, a contact imaging architecture was achieved using a white 5 mm LED as the light source, human cells adhered on tissue culture-coated glass substrates of various thicknesses, and a Charge-Coupled Device (CCD) or a Complementary Metal Oxide Semiconductor (CMOS) image sensor remotely controlled by their respective electronic devices (Fig. 1a).

The emission spectrum of the LED was characterised using spectrometry (Figure S1a). The emitted white light was mainly composed of a 465 nm blue peak supplied by GaN layers in the LED chip and a 565 nm green-yellow peak very likely produced by cerium-doped yttrium aluminium garnet (YAG:Ce) phosphor pigments. The emission spectrum ranges from 410 nm to 780 nm. Therefore, the LED emits no ultraviolet radiation which could induce cellular DNA alteration and no infrared light which could heat cell medium. The half-angle of the LED was measured by goniospectroradiometry to be 10.1° (Figure S1b). The LED was placed at about 5 cm above the image sensor to illuminate an approximate diameter of 18 mm at maximum radiation, far larger than the imaging areas of the employed image sensors. The produced illumination beam has limited temporal and spatial coherences as no optical filter nor pinhole were placed between the LED and the cell-supporting glass substrates.

The glass lids of colour and monochrome CCD image sensors were removed by local heating to enable a direct access to the pixels of the image sensors. Glass substrates covered with cells were then deposited onto the pixel area of the image sensors to record holograms from the cells at various distances from the image sensors, including at zero and very short distances (Figure S2a). Rough contact of the glass substrates with the pixel area may cause damages to the pixel architectures and consequently fatal failure of the image sensor. Additionally, the imaging surface got frequently covered with small glass debris detached from the thinnest, most fragile glass substrates used. To achieve this study, image sensors were unsoldered from their electronic daughterboards and replaced by sockets with the same pin pitch as the image sensors (Figure S2b). Defective image sensors plugged in the sockets could thus be rapidly substituted.

To maintain viable conditions for the cells, some cell culture medium was pipetted onto the image sensor dies previously to the deposition of the cell-covered glass substrates. As voltage difference between the pads and the bonding wires of image sensors was shown to generate an electrolysis of water present in the cell medium, every metal part of the image sensor packages was covered with the epoxy glob top Vitralit 1690 to separate the electrical connections of the image sensors and the liquid medium (Figures S2a, S2c). 35 mm-large bottom-pierced Petri dishes were glued with epoxy resist OG116-31 over the image sensor packages to provide millilitre-range culture chambers for the cells. Selection of the epoxy adhesives Vitralit 1690 and OG116-31 for the cell imaging device resulted from proliferation assays performed with human Retinal Pigmented Epithelial (RPE1) cells in the presence of various adhesives to assess their respective cytotoxicity (Figure S3).

Glass substrates with thicknesses *h* ranging from 50 μm to 1000 μm were diced and coated with fibronectin for cell adherence on one side and with anti-adhesive poly(L-lysin)-*grafted*-poly(ethylene glycol) (PLL-PEG) on the other side (Fig. 1a). Double functionalisation of glass substrates was achieved by PLL-PEG grafting on both sides of glass samples, removal of PLL-PEG from the top side using UV-ozone cleaning, and fibronectin grafting onto the top uncoated side. The glass substrates were then seeded with RPE1 cells, Human Bronchial Epithelial cells (16HBE), cervical cancer cells (HeLa) and Prostate Cancer cells (PC3) in Petri dishes at 37 °C in a CO_2_ incubator. The cell-covered glass samples were placed on the dies of image sensors with cell culture medium to acquire images of the cells.

Contact imaging of cells with non-coherent illumination (Fig. 1b) provides comparable images to phase contrast microscopy (Fig. 1c). The number, position and morphology of the cells appear to be similar. Cells are observed to spread and possibly form clusters with other cells according to the well-known behaviour of their respective cell line. RPE1 cells spread and remain mostly individual (Fig. 1b). 16HBE cells are assembled in small groups and are individually distinguishable within the clusters (Figure S4). HeLa cells have a slightly triangular shape. PC3 cells retain a round shape even in interphase and tend to form sparse clusters.

The holograms of the cells generated by contact imaging are bright objects with a white value higher than the background, resulting from both diffraction and focalisation of light by the cells (Fig. 1b, Figure S4). The contours of holograms are made of a single dark fringe surrounded by a single low- or medium-intensity bright fringe as a result of the limited coherence of the illumination.

### Ray tracing simulations and cell observations

Human cells have a diameter around 20 μm, *i.e.*, very large dimensions compared to the wavelengths of visible light. Ray tracing could thus be exploited to simulate the interactions of incident light with the cells.

**Figure 2:**
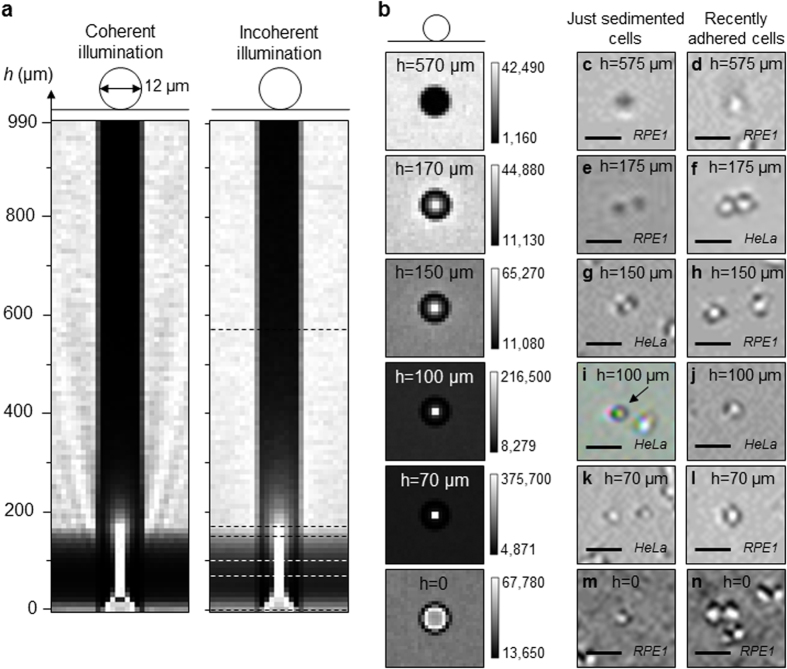
Ray tracing simulations and unprocessed contact images of round cells positioned at various heights *h* from the image sensor. (**a**) Intensity profiles collected by a row of 1.4 μm pixels after the rays go through a sphere of 12 μm in diameter and with a refractive index of 1.38 representative of a round cell. Each horizontal line indicates the radial intensity profile received by the image sensor under coherent and incoherent illuminations. (**b**) Ray tracing simulations through spherical cells under incoherent illumination. (**c**-l) Observations of round RPE1 and HeLa cells on tissue culture treated glass slides under incoherent illumination using monochrome and colour image sensors. The *just sedimented* cells (**c,e,g,i,k**) were individually identified in image sequences as stationary cells recorded *immediately or a few seconds* after sedimentation and rolling down to arrest. The *recently adhered* cells (**d,f,h,j,l**) were identified in image sequences as non-moving but still round cells *a few minutes* after cell seeding. m,n) Round RPE1 cells deposited directly on the pixel array of the image sensor. Scale bars: 50 μm.

**Figure 3:**
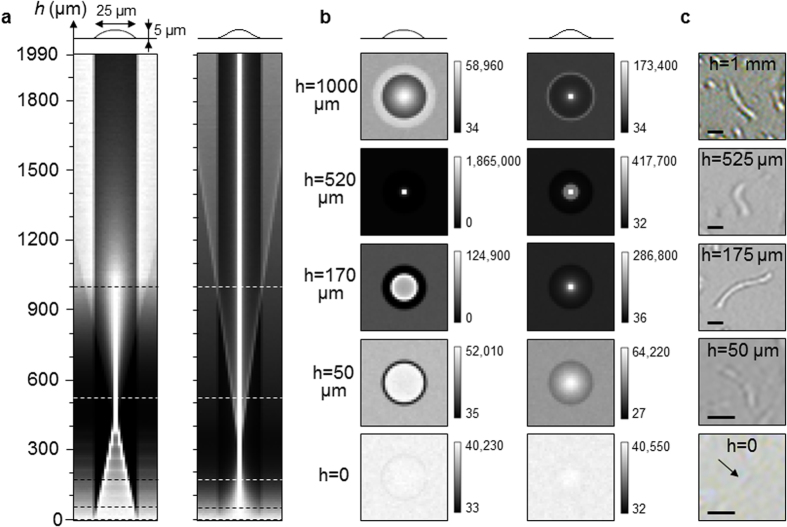
Ray tracing simulations and unprocessed contact images of adherent cells in interphase at various heights *h* under incoherent illumination. (**a**) Intensity profiles collected by a row of 1.4 μm pixels after the rays from an incoherent source go through a portion of a sphere (*left*) or a fourth degree polynomial (*right*) of 25 μm in diameter, 5 μm in height and with a refractive index of 1.38 representative of a spread cell. (**b**,**c**) Ray tracing simulations of spread cells (**b**) and observations of RPE1 cells in interphase with monochrome and colour image sensors (**c**) at 5 typical heights *h*. Scale bars: 50 μm.

RPE1 cells were used as the model cell line for the ray tracing simulations. The typical dimensions of RPE1 cells were measured using Scanning Electron Microscopy (Figure S5). Both cells in mitosis and in interphase were observed. The diameter of round mitotic RPE1 cells was measured on 12 cells to be between 10 and 13 μm. As a result, a diameter of 12 μm was employed for the ray tracing simulations of round cells (Fig. 2a,b). RPE1 cells in interphase show various bidimensional shapes. Spread RPE1 cells have a width between 12 and 55 μm with lamellipodia extending up to 30 μm, and a thickness between 3 and 6 μm at the position of the nucleus. The model cell used in the ray tracing simulations for the interphasic cell was thus defined with a diameter and a thickness of 25 μm and 5 μm, respectively (Fig. 3a,b). Moreover, dispersion in the shape of interphasic cells is common within a same cell culture. Spread cells were thus represented here by two shape models, a portion of a sphere and a fourth degree polynomial.

The mean refractive index of a cell depends on the refractive index and concentration of its components (cytoplasm, nucleus, mitochondria and proteins). The mean refractive indices of cells were measured to be 1.36 for erythrocytes^51^, 1.371–1.427 for MDCK cells^52^, 1.377 for neurons^53^, and 1.390–1.401 for tumour cells (Jurkat, HeLa, PC12, MDA-MB-231, MCF-7)^54^. Consequently, the cells in the ray tracing simulations were modelled with a mean refractive index of 1.38. Cell medium is mainly composed of water and was thus simulated with a refractive index of 1.33. The glass slides holding the cells have a refractive index of about 1.54.

We experimentally measured that the meniscus formed at the upper surface of the cell medium in a 35 mm Petri dish has a minor effect on the produced images, with a variation in the dimensions of the cells inferior to 3% depending on the shape of the meniscus. Refraction at the meniscus interface was thus neglected in the simulations.

Ray paths were calculated for cell-modelling objects positioned on a glass substrate whose thickness was varied from 0 μm (no substrate, *i.e.*, cells directly deposited on the image sensor surface) to 990 μm or 1990 μm for round and spread cells, respectively, with a vertical pitch of 10 μm (Figs 2b and 3b). As simulated images of the cells have circular symmetry, an intensity profile was extracted from every image and piled up in a graph to provide comparisons between images (Figs 2a and 3a). The minimum and maximum values of the lookup tables (LUT) were not normalised among the stack of images and in the piles of intensity profiles in order to preserve image contrast at every distance *h*. Non-normalisation of the LUTs is supported by experimental practice: when a cell culture is visualised, intensity of illumination is typically adjusted by the user to enhance contrast for a given distance between the cells and the image sensor.

Simulations of the refraction of incident light by round cells provide close results for both coherent and incoherent illuminations (Fig. 2a). The spherical cell acts as a ball lens focusing the rays into a photonic nanojet, *i.e.*, a thin beam emerging from the sphere with high intensity (Fig. 2a,b, h = 70 μm and h = 100 μm)^55^,^56^,^57^. The photonic nanojets under coherent and incoherent illuminations have maximum intensity at h = 60 μm and h = 50 μm and a full width at half maximum (FWHM) of about 57 μm and 70 μm along the optical axis, respectively. Transverse intensities of photonic nanojets are distributed on 2 adjacent pixels as a result of the 1.4 μm pixellisation in the model of image sensor.

Due to photonic nanojets, four types of images are obtained depending on the distance *h* between the round cell and the image sensor (Fig. 2a,b). For a distance *h* rising from 0 to ~30 μm, the rays are increasingly focalised into the photonic nanojet, and the round cell appears as a bright centre with a widening dark contour. For a distance between ~30 μm and ~100 μm, *i.e.*, inside the photonic nanojet, the refracted beam is focalised in a small area with intensity so high than the background appears to be dark comparatively. At distances *h* rising from ~100 μm to ~250 μm, the intensity of the photonic nanojet decreases so that the cell is imaged as a small bright centre surrounded by a large dark contour. At distances h >250 μm, the refracted beam is mainly diffused in the background.

Additionally, with coherent illumination, Zemax simulations show alternated bright and dark concentric rings surrounding the objects at distances h >80 μm (Fig. 2a). The alternating rings are positioned further from the optical axis as *h* increases. They were also revealed in previous studies of the photonic nanojets made by transparent dielectric microspheres under coherent illumination^56^,^57^. The concentric rings are particularly apparent in Fig. 2a because of non-normalisation of intensity profiles in the computational domain so as to preserve image contrast. With incoherent illumination, only a diffuse moderately bright ring is visible around the central pattern (Fig. 2b, h = 150 μm and h = 170 μm).

Ray tracing simulations are consistent with experimental observations of cells in suspension and just sedimented cells (Fig. 2c,e,g,i,k,m, Figure S6). As the length of photonic nanojets slowly varies with the microsphere diameter^55^,^56^, both round RPE1 and HeLa cells, which have close diameters, were imaged. Cells in suspension were identified in image sequences as objects moving at constant velocities in the medium. Just sedimented cells were identified in image sequences as objects rolling on the substrate surface at decreasing velocities down to arrest. The cells sedimented directly on the pixel array of the image sensor (h = 0) or at a distance h = 70 μm from the image sensor are visualised as bright quasi-circles with a dark contour (Fig. 2k,m), confirming that the image sensor is placed at the beginning and within the photonic nanojets produced by these spherical cells, respectively. At h = 100 μm and h = 150 μm (Fig. 2g,i), the cells are imaged as bright centres with a dark and larger contour than in the photonic nanojet (Fig. 2k) and with a diffuse external bright contour, showing that the image sensor is placed at the end of the photonic nanojets produced by the spherical cells. Finally, at h = 175 μm and h = 575 μm (Fig. 2c,e), round cells are observed as dark shadows with a diffuse bright contour, in agreement with the diffusion of the refracted beam into the background.

The cells imaged in Fig. 2e are different from ray tracing simulations at h = 170 μm (Fig. 2b) as a likely result of the approximations employed in the calculations, *e.g.*, a round cell modelled as a homogeneous sphere. The transition between the bright pattern in the photonic nanojet and the dark pattern far from the photonic nanojet occurs at distances *h* in the 150–175 μm range. Consequently, the ray tracing simulations are in very good qualitative agreement with experimental observations for the just sedimented cells.

Cells in suspension produce similar patterns (Figure S6). When the cells in suspension are enabled to flow very close to the image sensor, including at a distance h < 150 μm, *e.g.*, by positioning a 50 μm-thick glass substrate above the image sensor, a few flowing cells are visualised as bright objects with a dark contour while most of the cells are seen as uniformly dark patterns (Figure S6a), very likely depending on their distance to the image sensor. The observation of cells in suspension as bright patterns confirms the ability to record the photonic nanojets produced by cells in suspension at small distance from the image sensor. Transient patterns between the bright and dark patterns are also observed (Figure S6b), certainly corresponding to the end of the photonic nanojet. On the opposite, when the cells in suspension are maintained at a distance h ≥ 175 μm, *e.g.*, by positioning a 175 μm-thick glass substrate above the image sensor, the cells in suspension are exclusively observed as dark patterns (Movie S1). The dark patterns of cells in suspension at h ≥ 175 μm are larger and have diminishing contrast compared to the background for growing distances *h* to the image sensor as a result of the illumination cone.

Cells recently adhered on fibronectin-coated glass slides were identified as non-moving but still round objects in image sequences acquired a few minutes after cell seeding. Recently adhered cells display a bright pattern with a dark contour at every distance *h* (Fig. 2d,f,h,j,l,n). An external diffuse bright contour is also observed at h > 0. Furthermore, PC3 cells in interphase retain a round shape and could thus be used to investigate the patterns of distant adhered round cells (Figure S4c, Figure S7). Similarly, round adhered PC3 cells are visualised as bright circles with a dark contour and a diffuse bright contour at distances *h* between 505 μm and 800 μm. This homogeneous pattern for every distance *h* is significantly different from the one of just sedimented cells and cells in suspension at distances h ≥ 175 μm (Fig. 2c,e, Movie S1), suggesting that refraction of light by quasi-round adhered cells meaningfully differs from the ball lens model. Although recently adhered cells display a seemingly round pattern, the tridimensional shape of adhered cells may diverge from the sphere during the adhesion process, possibly as a result of binding of the cell membrane to the extracellular matrix (ECM) secreted by the cell.

The two shape models used to represent spread cells, a portion of a sphere and a fourth degree polynomial, provide different intensity profiles (Fig. 3a,b). As ray tracing simulations supply comparable results for a particular shape model using coherent and incoherent incident lights, only simulated images and intensity profiles with non-coherent illumination are displayed in Fig. 3a,b. Light is focalised at approximate distances of 450 μm and 250 μm for the simulated spherical lens-like and aspheric lens-like shapes, respectively. Refraction of the incident light by spread cells is thus very dependent on cell morphology. As long as the culture is nonconfluent, the tridimensional shape of an interphasic cell is ever changing over time in relation to its motility behaviour, inner mechanical forces produced by the cytoskeleton and osmotic pressure, its biochemical environment, local adherence on the ECM and cell-cell contact, which implies that light refraction by a cell is continually changing over time. In addition, as previously suggested by the pattern difference between just sedimented cells and adhered quasi-round cells at h ≥ 175 μm (Fig. 2c–f), even a small tridimensional change in the cell shape may significantly modify refraction of light by the cell. As the distribution of tridimensional morphologies of interphasic cells is usually large within the cell culture, light focusing behaviours at any given time are very diverse among the cell population.

Nevertheless, contact imaging of RPE1 cells at several distances h > 0 shows that the cells can be visualised with a relatively satisfying resolution on the whole tested range of distances (Fig. 3c). These observations significantly differ from geometrical optics simulations at the similar distances *h* (Fig. 3a,b), which emphasises great sensitivity of light refraction to cellular morphology.

Interestingly, at a distance h = 0, *i.e.*, when the cells are in direct contact with the surface of the image sensor, the contrast of the object is very low compared to the background for both simulations (Fig. 3b). This feature is very consistent with experimental observations of spread RPE1 cells for which the cells are almost undistinguishable from the background (Fig. 3c, h = 0). On the opposite to spread cells, refraction of ball lens-like cells at the distance h = 0 provides images with good contrast (Fig. 2m,n).

**Figure 4:**
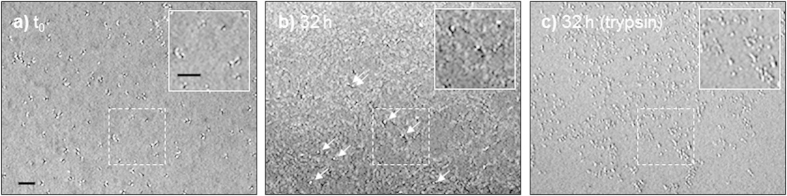
RPE1 cells on the surface of the image sensor (h = 0) under incoherent illumination. (**a**) Image taken a few minutes after cell deposition when RPE1 cells just adhered on the surface. (**b**) Visualisation of the cells after 32 h in an incubator. White arrows show spherical mitotic cells. (**c**) Same cells at 32 h after trypsinisation. Scale bars: 100 μm.

Contact imaging at zero distance between the cells and the image sensor was specifically examined by pipetting RPE1 cells onto the imaging area and culturing them for 32 h in a CO_2_ incubator (Fig. 4). Recently adhered cells are still spherical and can be actually detected on the recorded image under non-coherent illumination (Fig. 4a) in a similar manner to Fig. 2n. After cell spreading on the image sensor, the presence of the cell culture is revealed by the increased surface roughness appearing on the image (Fig. 4b). Spread cells are difficult to distinguish from the background, in accordance with ray tracing simulations and previous observations (Fig. 3b,c, h = 0). On the opposite, rounded-out mitotic cells, highlighted by white arrows in Fig. 4b, produce a distinct pattern which is comparable to the one of round cells in Figs 4a and 2n. Consequently, only mitotic cells distinctly emerge from the background at h = 0 during incubation. The two top arrows in Fig. 4b show two daughter cells likely just after cytokinesis. Finally, cells were detached from the surface using 0.05% trypsin/EDTA so that all the cells take a round shape and become refractive once again (Fig. 4c). The number of cells has increased by a factor of ~2.4 within 32 h as a result of proliferation on the surface of the image sensor.

### Sedimentation, rolling and attachment

**Figure 5:**
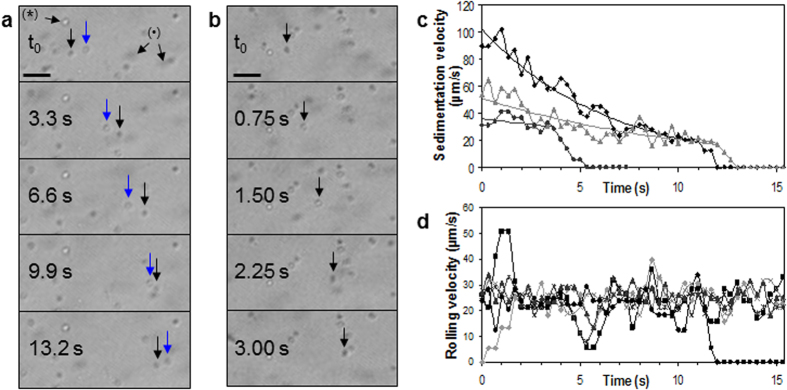
Sedimentation, rolling and attachment of RPE1 cells onto a fibronectin-coated glass substrate (h = 175 μm). (**a**) Cells in suspension (two examples are marked by the sign •) are observed as moving dark patterns whose sharpness progressively increases as the cell approaches the surface. Sedimented cells which likely have started their adhesion process (an example is marked by the sign *) appears to have a bright body with a dark circular contour. The two vertical arrows show cellular rolling and adhesion. The black arrowed cell has just begun sedimenting onto the surface at t_0_ and is progressively losing its velocity until definitely stopping at the end of the image sequence. The blue arrowed cell previously tethered to the surface rolls further under shear flow. Scale bar: 100 μm. (**b**) A sedimenting cell transiently showing a bright pattern during deceleration on the surface. Scale bar: 100 μm. (**c**) Velocity of cells previously in suspension during their sedimentation process onto the fibronectin-coated glass surface. (**d**) Velocity of six recently deposited cells rolling on the surface under shear flow. The velocity reduction simultaneously affecting several cells at 5.5s is likely due to a transient decrease in shear flow.

The possibility to monitor sedimentation, rolling and adhesion of cells on a substrate using contact imaging with non-coherent illumination was demonstrated. A suspension of RPE1 cells was pipetted onto a 175 μm-thick fibronectin-coated glass substrate positioned on the pixel area of a CCD image sensor. The sedimentation, rolling and attachment of cells were filmed by the image sensor at 30.0 frames per second (Fig. 5a, Movie S1). The motions of the cells in suspension reveal the movement of the medium within minutes after deposition with the pipette. The cells in suspension are observed as dark objects moving rapidly throughout the imaging area, *i.e.*, the typical pattern produced by spherical cells far from the photonic nanojet. The contrast of the cells in suspension is low when their distance *h* to the image sensor is high. Conversely, their hologram becomes sharper when the cells get closer to the substrate surface (h = 175 μm).

When a cell in suspension touches the fibronectin-coated glass slide, it progressively loses its velocity by rolling on the surface until attaining firm arrest (Fig. 5a, black arrowed cell). The velocity of cells decreases as a result of friction produced by molecular interactions transiently formed between the cell membrane and the fibronectin coating. Deceleration of the sedimenting cells follows an exponential decay with measured exponential decay constants ranging between 0.047 and 0.156 s^−1^ on the fibronectin-coated glass surface (Fig. 5c). During deceleration, sedimenting cells mainly display the typical uniformly dark pattern, but in some cases also transiently shows a bright pattern with a dark contour while still rolling on the fibronectin-coated surface (Fig. 5b). The latter behaviour was observed in 45% of cell sedimentations and frequently lasts less than 0.5 s before returning to the dark pattern, which suggests that the sedimenting cell is briefly deformed from the sphere by molecular binding with the fibronectin coating before restoring its spherical shape when chemical bonds are broken.

When they are finally stopped on the surface, just sedimented cells display a dark pattern similar to Fig. 2e, and after a few minutes produce a bright pattern with a dark circular contour similar to Fig. 2f as a likely result of a modification of the cell shape by, *e.g.*, cell binding to the newly secreted extracellular matrix. The transition from a dark pattern to a bright pattern of the just sedimented cells as a likely result of cell-substrate adhesion, observed here using an incident light of both limited temporal and spatial coherences, is in strong agreement with previous reports using coherent illumination^39^.

Moreover, the just sedimented cells are poorly attached to the surface and consequently can be pushed further by the shear flow (Fig. 5a, blue arrowed cell). Cells rolling under the shear flow are only visualised by a bright pattern, suggesting that molecular interactions continuously deform the cell in its path. Cells roll on the surface until being slowed down and stopped by sufficiently strong interactions with the surface coating. The measured average rolling velocity is 25.1 ± 4.0 μm s^−1^ (Fig. 5d). This rolling velocity is close to those reported on surfaces coated with other cell adhesion molecules^58^,^59^.

When the velocity of sedimenting cells decreases down to the range of the average rolling velocity, they tend to continue rolling a few seconds at that velocity and then abruptly decelerate until arrest (Fig. 5c). The rolling and arrest behaviour of sedimenting cells is similar to that of cells rolling under shear flow (Fig. 5d). The sharp deceleration observed during the arrest phase (Fig. 5c) certainly results from a sudden increase of the effect of bonds formed between the cell and the fibronectin coating. The acceleration and deceleration of rolling cells (Fig. 5d) and deceleration of sedimenting cells in their arrest phase (Fig. 5c) are measured to last 1.26 ± 0.66 s on fibronectin-coated glass under the pipette deposition conditions.

### Adhesion, proliferation and motility

**Figure 6:**
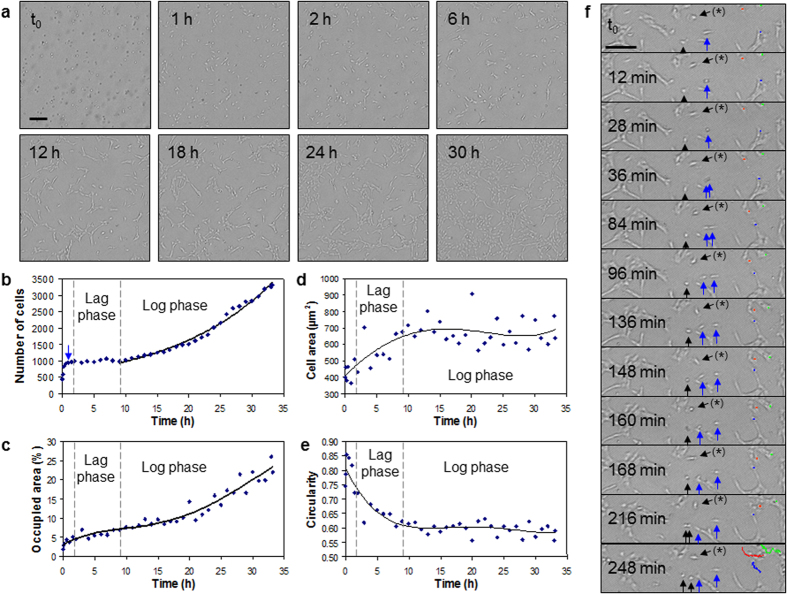
Adhesion, proliferation and motility of RPE1 cells on a fibronectin-coated glass slide (h = 175 μm). (**a**) Adhesion, spreading and proliferation of RPE1 cells. The cell areas first increase while the cells spread on the surface. Mitosis and displacements of the cells can be individually followed up. (**b**) Adhesion and proliferation of the cell population. The vertical dashed lines separate the seeding (*left*), lag and log phases. The arrow indicates the opening and closing of the incubator door 40 min after cell seeding (see Figure S8). An exponential curve fits the population growth in the log phase. (**c**) Measurement of confluence, *i.e.*, percentage of the imaging area occupied by the cells. (**d**) Average area occupied by a cell. (**e**) Average cell circularity. (**f**) Cell divisions and migrations. Two cells (vertical arrows) successively divide into daughter cells. With images acquired every 4 min, the first cell is observed with a bright pattern during mitosis, while the second one is transiently dark at t = 160 min. Tracking of the three single cells marked with a colour point on every image is shown in overlay lines at t = 248 min. The cell labelled by the sign (*) presents distinctive nucleus and lamellipodium. Scale bars: 200 μm.

Growth of cultures of RPE1 cells was monitored from the deposition of the cells to near confluence on a fibronectin-coated glass substrate (h = 175 μm) using contact imaging with a pierced Petri dish employed as a culture chamber and under illumination of limited coherence (Fig. 6a, Movie S2). The cable connecting the cell imaging device in the CO_2_ incubator and the computer was pressed against the seal of the incubator door or passed through the access port on the rear panel of the incubator to provide sterile conditions for the culture.

Both cells in suspension and sedimented cells are observed on the same image a few minutes after deposition of the cell suspension with a pipette (Fig. 6a, time t_0_). The number of cells rapidly increase as cells sediment on the glass substrate and finally stabilise within the first 90 minutes (Fig. 6b). During this sedimentation and attachment phase, the first deposited cells adhere and spread on the surface (Figs 6a, 1h), which results in an increase of the average cell area (Fig. 6d) and a decrease of the average circularity index (Fig. 6e). The confluence of the cell population increases due to both sedimentation of cells from the suspension and spreading of adhered cells (Fig. 6c).

A lag phase is then observed in which cells adapt themselves to growth conditions but do not divide. No significant variation in the amount of cells is recorded for ~7.5 hours (Fig. 6b). During this period, the cells increasingly spread on the surface (Fig. 6a, 2h and 6h compared to 1h), resulting to rising total occupied area (Fig. 6c), rising average cell area (Fig. 6d) and diminishing average circularity (Fig. 6e).

The RPE1 cell population then enters the log phase, in which the number of cells increase exponentially (Fig. 6b). A doubling time of 13.9 h was measured in the log phase, indicative of RPE1 cells proliferating rapidly in good culture conditions^60^. No reduction in cell growth is observed within 24 h as a result of the large volume of medium offered by the 35 mm Petri dish. Cell occupancy grows from 7% to 25% within 24 hours (Fig. 6a, 12 h–30 h; Fig. 6c). Cell occupancy in the log phase is linearly correlated with the number of cells with a coefficient of determination R^2^ = 0.91, showing that evolution of confluence is mainly driven by the growth of the cell population and not anymore by cell spreading. This is confirmed by the average cell area and circularity values which are almost constant in the log phase at 677 ± 81 μm^2^ and 0.60 ± 0.02, respectively, showing that the cells have reached maximum spreading and present a steady morphology at the population scale (Fig. 6d,e). The average cell area determined from contact imaging is consistent with those measured from SEM characterisation (Figure S5 and dimensions reported in the section “Ray tracing simulations and cell observations”).

The blue arrow at 40 min in the seeding phase denotes the gentle opening and closure of the door of the incubator (Fig. 6b). While the evolution of the population seems unperturbed in Fig. 6b, a small fraction of the cells was detached and returned back in suspension as a result of the mechanical vibrations produced by the incubator door (Figure S8). The detached cells are visible as dark objects whose number rapidly decrease within the first 10 minutes (Figure S8a). Some cells sediment on the surface until ~35 min after the door opening. Typical changes of the holographic patterns of the cells, from dark objects to bright patterns with a dark contour, are observed when cells adhere to the fibronectin-coated surface (Figure S8b). Detachment of just adhered cells even by gentle handling of the incubator door highlights the precarious situation of adherent cells in the seeding phase.

Mitosis and migration of individual cells can be monitored (Fig. 6f, Movie S2). Cells entering into mitosis round up and become brighter than spread cells^35^,^36^. Focalisation of light by round mitotic cells is typical of the refringence phenomenon also experienced with optical microscopy, *e.g.*, phase contrast microscopy. Using time-lapse contact imaging with an image acquisition frequency of 4 min, most of dividing cells are observed with a bright pattern during the whole process (blue arrow in Fig. 6f). In a few instances, the dividing cells are visualised as transiently dark patterns (black arrow in Fig. 6f). This dark pattern is produced by perfectly spherical cells far from the photonic nanojet (Fig. 2e), and may reveal a temporary modification of cell adhesion to the ECM lasting a few tens of seconds at metaphase. After cytokinesis, the two daughter cells move away from one another and take the specific morphology of the cell line.

RPE1 cells in interphase were tracked to determine the typical velocities for this cell line (Fig. 6f). The cells moved randomly on the substrate due to the absence of any chemoattractant. Most RPE1 cells migrated at velocities ranging between 0.5 and 1.6 μm/min. Fastest RPE1 cells have been observed to move at 1.8 μm/min. These motility values are typical of human cell lines.

No intracellular feature is visible in most observations. However, in one case, the nucleus appeared to be more refringent than the cell as a whole (Fig. 6f). In the cell labelled by the sign (*), the nucleus and the lamellipodium are distinctly apparent at t_0_. In the following images, this cell moves back-and-forth around its initial position. The nucleus and the lamellipodium are clearly distinctive at the times 36 min, 84 min, 160 min, 168 min and 216 min. This unique optical behaviour suggests that distinguishing images of a cell can be generated as a likely result of independent light focalisation and diffraction by both the nucleus and the cell membrane.

**Figure 7:**
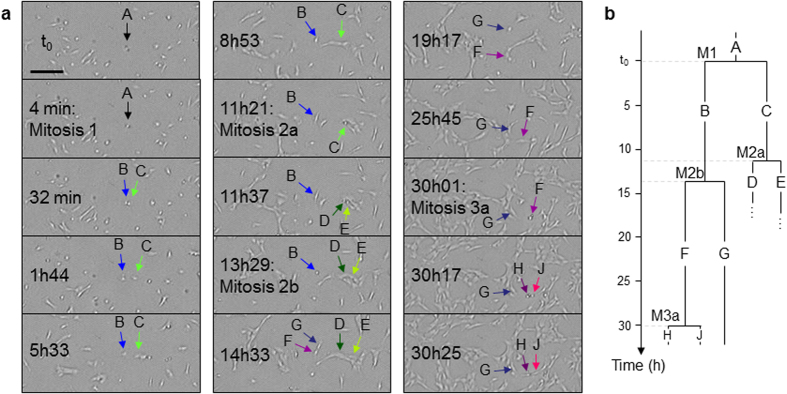
Lineage of a RPE1 cell on a fibronectin-coated glass slide (h = 175 μm) for 32 hours. (**a**) Successive divisions of cells originating from the same cell *A*. The branch through the cell *B* could be established along 4 generations. On the opposite, the cells *D* and *E* could not be continuously tracked within the dense cluster of cells where they were formed. Scale bar: 200 μm. (**b**) Lineage of the cell *A*. Mitoses are labelled M*x* where *x* = 1, 2a, 2b or 3a refers to the corresponding mitoses in (**a**).

Lineage of individual cells can be established by monitoring successive mitoses and movements of daughter cells (Fig. 7). Successful cell tracking for a period of tens of hours requires frequent image acquisitions, *e.g.*, every 5 min, and a minimum distance of ~20 μm between the monitored cells and the neighbouring cell clusters so that cells can be individually identified. The progeny of a RPE1 cell until the fourth generation could be observed for 32 hours (Fig. 7). The average duration between two successive divisions in this lineage is 13.8 h, in strong agreement with the doubling time of 13.9 h previously measured in the log phase for the whole cell population (Fig. 6b). In Fig. 7, some third-generation daughter cells could not be tracked as they became undistinguishable from neighbouring cells in a cluster.

### Heat-induced cell death

**Figure 8:**
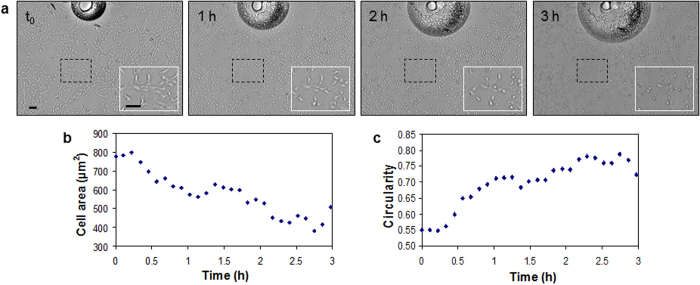
Heat-induced cell death. (**a**) RPE1 cells on a fibronectin-coated glass slide (h = 175 μm) are exposed to a temperature of 52 °C. When heating is triggered, the cells rapidly round up. The bubble at the top of the image grows due to heating and pushes the cells nearby, showing that the cells are detached from the surface. Scale bars: 100 μm. (**b**) Average area occupied by a cell. (**c**) Average cell circularity.

The RPE1 cell population monitored in the Movie S2 was then exposed to a temperature increase up to 52 °C to visualise cell death (Fig. 8, Movie S3).

At time t_0_ when heating is triggered, RPE1 cells present their typical elongated morphology (Fig. 8a). The temperature raised from 37 °C to 50 °C in 4 minutes, then stabilised at 52 °C after 12 more minutes. RPE1 cells exposed to overheat have an unchanged morphology for the first 12 min (Fig. 8b,c) as a likely result of thermal inertia of the medium. Then, cells contract and continuously round up. Values of average cell area and average cell circularity are linearly anticorrelated with a coefficient of determination R^2^ = 0.93. The cells which were in mitosis phase at t_0_ stop dividing and remain round thereafter (Movie S3). The number of cells is unchanged during overheating exposure. Cells located in clusters at t_0_ become individually distinguishable within the first 30 minutes as a result of reduction of cell areas.

Additionally, a bubble present on the top part of the image is growing with the temperature increase (Fig. 8a, Movie S3). The expanding bubble pushes the neighbouring cells, which demonstrates that the cells are not attached to the surface anymore and have most likely become cell debris. Interestingly, the patterns of dying RPE1 cells remain bright in our observations, while those of other cell lines were reported to become dark using coherent illumination^39^, suggesting that different cell death processes may be characterised by distinctive pattern dynamics.

## Discussion

### Object beam shaping by cells

Both geometrical optics simulations and experimentations with human cells were used to investigate the contribution of refraction by the cells themselves to the produced diffraction patterns of the cells. Light refraction by cells significantly varies during the adhesion process and along the cell cycle as a result of modifications in the tridimensional morphologies of the cells. As the cells are constantly adapting their shape in response to inner mechanical forces and their environment, light refraction evolves accordingly over time.

Cells in suspension, cells just deposited on a transparent substrate, mitotic cells as well as cells rounded-out by trypsin were shown to focalise light into photonic nanojets, *i.e.*, thin beams with very high intensity, as a result of their spherical shape in a similar way to transparent dielectric microspheres (Fig. 2c,e,g,i,k,m, Figs 4b,c, 5a and 6a,f, Figure S6, Figure S8).

The photonic nanojets generated by round cells can be recorded when the image sensor is placed at a distance h ≤ 150 μm from the cells. This situation was achieved by removing the glass lid covering commercial image sensors and placing a thin tissue culture-treated transparent substrate (Fig. 2g,i,k, Figure S6) or depositing the cells directly on the pixels of the image sensor (Figs. 2m and 4). However, this latter approach prevents distinguishing adherent cells spread on the surface (Fig. 4b). To visualise the photonic nanojets of cells in suspension, the flowing cells certainly have to be maintained at a maximal distance of 150 μm from the image sensor, *e.g.*, by placing a top glass slide above the cells or using a microfluidic channel or microchamber with a wall thickness equal to or less than 150 μm.

At a distance h ≥175 μm, round cells are visualised by a uniformly dark pattern (Figs 2c,e, 5a and 6a,f, Figure S6a, Figure S8) corresponding to the end of the photonic nanojet as the beam refracted by spherical cells is mainly diffused in the background (Fig. 2a,b). A position of round cells, especially cells in suspension, below or above the transition height h = 150–175 μm can thus be easily detected on the images based on the cell patterns. Additionally, distant round cells, *e.g.*, cells in suspension, illuminated with a non-collimated incident light display dark patterns of diminishing contrast for growing distances *h* (Fig. 5a, Movie S1).

The length of photonic nanojets linearly but slowly increases with the diameter of the sphere, *i.e.*, with a small coefficient of approximately 0.2.^55^,^56^ As a consequence, the transition height h = 150–175 μm was experimentally determined using both round RPE1 and HeLa cells as a result of the close diameters of these human cells (Fig. 2c,e,g,i,k,m). Most animal cells have dimensions in the 10–100 μm range. As a result of the slow increase in the length of the photonic nanojets with the diameter of round cells, we suggest that, in addition to RPE1 and HeLa cells, the uncovered transition height h = 150–175 μm between patterns of round cells may be a valuable order of magnitude for many other animal cell types.

In our ray tracing simulations, spread cells were modelled by spherical and aspheric plano-convex lenses (Fig. 3a,b). These shapes appeared to be too simple to model light refraction by spread RPE1 cells as the simulated focalisation distances were not validated by experimental observations (Fig. 3c). An exception is the low contrast between spread cells and the background when the cells have adhered to the image sensor surface (Fig. 3 h = 0, Fig. 4b). For geometrical optics simulations to appropriately predict refraction by spread cells, the accurate tridimensional morphology of the cells has first to be determined, *e.g.*, using confocal microscopy, SEM and focused ion beam (FIB) sectioning.

The cells in culture certainly refract light in diverse ways as a result of the variety of morphologies of adhered cells in interphase. However, different cell lines exhibit distinctive morphologies, *e.g.*, elongated, triangular and round shapes for RPE1, HeLa and PC3 cells, respectively (Fig. 1b,c, Figure S4). Consequently to shape constraints inherent to a cell type, interphasic cells might focalise light on a specific range, or distribution, of distances as well as in spots of specific sizes and shapes. Furthermore, we suggest that cells in interphase may focalise further over time, as the cell size progressively increases along the G_1_, S and G_2_ phases. The combined effect of cellular size and morphology result in various shapes of the object beams among the cell population. In a special case, some adherent cells in a gel, thus positioned at different distances *h*, may project similarly on the image sensor, but could be distinguished by distinct diffraction patterns in the object beams.

The transition events between the ball lens behaviour and the plano-convex lens behaviour, representative of round and spread cells, respectively, are of particular interest at a distance h ≥ 175 μm of the image sensor. As spherical cells at these distances display the very distinctive dark pattern generated far from the photonic nanojet, any change from or to the round shape can be easily detected. Cells sedimenting and decelerating on a coated surface were frequently observed to be momentarily deformed, as a very likely result of molecular interactions briefly formed between the cell membrane and the surface coating, before returning to a spherical shape (Fig. 5b). Cell distortion from the sphere when rolling on a coated surface was confirmed by the observation of non-adhered cells pushed further by the shear flow (Fig. 5a, blue arrowed cell). Furthermore, the transition between a dark pattern and a bright pattern is a signature of cell adhesion to the surface coating or secreted ECM (Fig. 2c–f, Fig. 6a, Figure S8b), as well as cells entering metaphase (black arrowed dividing cell on Fig. 6f at t = 160 min).

The extent of morphology modification required for this pattern change between round and spread cells is currently unknown. Brief bright patterns of decelerating cells during sedimentation (Fig. 5b) and continuously bright patterns of rolling cells (Fig. 5a, blue arrowed cell) suggest that they may be generated by modest shape changes. Interestingly, filipodia tethering the cells to the substrate during mitosis (Figure S5) do not affect the generation of photonic nanojets (Fig. 6f, t = 160 min).

### Contact microscopy under non-coherent illumination

In previous studies^23^,^26^,^31^,^32^,^37^,^38^,^39^,^40^,^41^,^42^,^43^,^44^,^45^, contact imaging of cells was produced using coherent, quasi-coherent or partially non-coherent illumination. This work demonstrates that brightfield contact imaging of cell cultures can be successfully obtained even using incoherent incident light produced by a white, directional LED with no optical filter, spatial filter nor holographic reconstruction. Despite blurring of interference patterns, contact imaging using an illumination of both limited spatial and temporal coherences was shown to provide efficient conditions for cell microscopy.

The image quality of cells is good in our imaging conditions for the four tested human cell lines, *i.e.*, RPE1, 16HBE, HeLa and PC3. The images of cells seemed unaffected by the presence of phenol red in the cell media, as possible absorption of light by coloured medium can be counterbalanced by slightly increasing the intensity of the light source.

Contact video microscopy with non-coherent illumination was successfully applied to several major applications in cell biology, including sedimentation, rolling and adhesion of cells on surface-coated substrates, division^35^,^36^, proliferation^47^,^48^ and lineage determination, monitoring of cellular morphologies, motility, death and detachment. These cellular phenotypes can be exploited to investigate environmental or drug effects on cell types. In addition, time-lapse contact microscopy can help uncovering previously unknown features of a cell line, *e.g.*, we have previously reported the tendency of the semi-adherent S2 cells from *Drosophila melanogaster* embryo to form vertical columns by aggregations or divisions, likely to minimise contact with the surface^47^,^48^.

Contact imaging typically provides larger fields of view than those allowed by microscope objectives, which is of particular interest to observe a representative cell population, *e.g.*, quantify cell motility as a significant fraction of the cells remain on the imaging area over a sufficiently long period of time. Image sensors providing very wide fields of view, *e.g.*, 50 × 37 mm commercial sensors, may be convenient to record the growth of especially large cells such as neurons without any image stitching. Additionally, monitoring of a large population on a long timescale may be used to detect infrequent cell events such as cell differentiation, and classify co-cultured cell types on the basis of distinctive cell size, morphology, motility or staining.

In this study, the cells were cultured on fibronectin-coated glass slides and imaged at distances from 0 to 1000 μm (Fig. 3c). Contact imaging in multiwell microtiter plates^47^,^48^, multiwell crystallisation plates^22^, Petri dishes^37^,^44^, microfluidic channels^46^ and in Matrigel^45^ was also reported. These results demonstrate the exceptional depth of field achieved by this imaging technique.

Conversely, under incoherent illumination, contact imaging of unlabelled cells rarely provides intracellular detail. The distinction of the nucleus and the lamellipodium in an adhered RPE1 cell is a noticeable exception (Fig. 6f), possibly resulting from an uncommon cell morphology.

Furthermore, resolution of holograms depends on the pixel size of the image sensor and the illumination cone. Considering the resolving power of the cell imaging devices being only due to the pixel size, the image sensors with 5.6 μm, 1.75 μm and 1.4 μm pixels used in this study provide numerical apertures (NA) of 0.04, 0.13 and 0.17 at the 465 nm blue peak (Figure S1a), respectively. For purposes of comparison, standard 4× microscope objectives have NA ~ 0.13. The produced raw images of the cell cultures are qualitatively close to those of 4× microscope objectives (Fig. 1b,c, Figure S4a).

Resolution of contact imaging can certainly be improved further in a close future in line with pixel downscaling. Top-of-the-art commercial image sensors currently have 1.1 μm pixels and the 0.9 μm pixel generation is under development^61^, while academic studies have reported innovative image sensors having 0.7 μm^62^ and even 0.5 μm^63^ pixel sizes.

Contact imaging with a white light reported here simplifies the experimental setup by reducing the specifications on the spectral width of the incident light and by avoiding the production of a secondary point source. Directly interpretable images are obtained without any numerical reconstruction step, which supports very fast recording speeds of the sample. This feature was exploited by the image acquisition of sedimenting and rolling cells on a transparent substrate at the frame rate of 30.0 images per second. Finally, the presented illumination configuration paves the way for direct colour imaging with a single image acquisition.

## Conclusions

Time-lapse contact imaging of live cells in a CO_2_ incubator was demonstrated using a light source of both limited temporal and spatial coherences. The glass lids covering the image sensors were removed to enable digital recording of human cells in suspension, just sedimented cells and adhered cells at distances between 0 and 1000 μm from the pixel array of the image sensors. Light refraction by cells was shown to significantly vary during the adhesion process, along the cell cycle of a particular cell and among the whole cell population in relation with the tridimensional morphology of every cell. Cells in suspension, cells just sedimented on a transparent substrate and rounded-out cells act as a ball lens focusing the rays into a photonic nanojet which can be visualised by positioning the image sensor at a distance inferior to 150 μm from the cells. Conversely, the object beam refracted by spherical cells is mainly diffused in the background for distances superior to 175 μm. Furthermore, temporary or stable molecular binding of cells rolling or adhered on the substrate coating provides significant changes in the tridimensional cell shape and consequently in the light focalisation properties of the cells. Distinct patterns for the nucleus and the lamellipodium can also be displayed in some favourable conditions. Contact imaging of cell cultures with non-coherent illumination can be used in many applications, including studies of cell sedimentation, rolling, attachment, spreading, proliferation, division, monitoring of lineages, motility, cell death and detachment.

## Materials and Methods

### Image sensors

Live-cell populations were imaged using colour (Bayer filter array) and monochrome ICX098 Charge-Coupled Devices (CCDs) from Sony (Japan) driven by a remote-control electronic board Fire-i from Unibrain (Athens, Greece). The CCD array is composed of 640 × 480 5.6-μm square pixels, thus supplying a 3.6 × 2.7-mm field of view. The image sensors were unsoldered from the daughterboards and replaced by 14-pin Dual In-line Package (DIP) sockets IS232-414 from Andon Electronics (Lincoln, Rhode Island, USA) covered by 1.27 mm pitch female connectors to enable substitution of image sensors possibly made faulty or dirty after the deposition of glass substrates onto them (Figure S2). The glass lids of image sensors were unglued in a clean room by locally heating the lids at 400 °C to get direct access to the dies of image sensors. The non-conductive and thixotropic UV curable glob top Vitralit 1690 supplied by Eleco Produits (Gennevilliers, France) was manually deposited onto the gold contact pads of the image sensor dies, on the pad area of the plastic packages and on the gold wires bonding the die pads and the package pads (Figure S2a). Reticulation of Vitralit 1690 during the deposition process was obtained by successive UV exposures for 30 s, followed by a final thermal annealing at 105 °C for 30 min. This glob top encapsulation prevented electrolysis of water present in cell medium as a result of the electric potential difference between metal parts of the image sensor. Every printed circuit boards and electronic components, except the image sensors, were enclosed in a home-made package to prevent possible damage from 100% relative humidity in the CO_2_ incubator. Additionally, mL-range cell culture chambers were produced by fixing 35 mm-large bottom-pierced Petri dishes onto the image sensor packages using UV curable epoxy adhesive OG116-31 from Epoxy Technology (Billerica, USA)^36^. The Petri dishes were covered with their lid in the CO_2_ incubator.

Colour (Bayer filter array) Complementary Metal Oxide Semiconductor (CMOS) image sensors VS6754 and VD6953 from STMicroelectronics (Grenoble, France) mounted on daughterboards were also used with a PCB1212 Rev. B generic capture board (STMicroelectronics). VS6754 and VD6953 image sensors have arrays of 1600 × 1200 1.75-μm and 2592 × 1944 1.4-μm square pixels, corresponding to 2.8 × 2.1-mm and 3.6 × 2.7-mm fields of view, respectively. The upper lens packaged above the image sensor VD6953 in a camera module was unscrewed to get access to the image sensor and position the cell cultures at various distances from the image sensor.

### Biocompatibility assays

As the cell culture medium was in direct contact with the die of image sensors and with the plastic package, ability of cells to adhere and proliferate with normal morphology in this environment was investigated. Retinal Pigmented Epithelial (RPE1) cells were pipetted into a CCD image sensor die and cultured for 16 h at 37 °C in a CO_2_ incubator. The cells were then rinsed twice with Phosphate-Buffered Saline (PBS), fixed with a 4% paraformaldehyde (PFA) solution in PBS for 15 min at room temperature and rinsed with PBS. To achieve immunofluorescence labelling of the cells, cell membranes were first permeabilised using 0.1% Triton in PBS. Cell nuclei and actin were labelled using 0.1 μg/mL Hoechst and 1 μg/mL phalloidin-FITC in PBS for 15 min at room temperature, respectively. The cell culture was finally rinsed with PBS and imaged using an epifluorescence microscope BX51M from Olympus. PBS was acquired from Life Technologies (Saint-Aubin, France), and every other chemicals from Sigma (Saint-Quentin Fallavier, France).

In addition, to select the two epoxy adhesives employed in the encapsulation process of the CCD image sensors, Vitralit 1690 and OG116-31, cytotoxicity of 8 epoxy adhesives was investigated by monitoring adherence, proliferation and morphology of RPE1 cells in the presence of a few millilitres of glue. The epoxy resists were deposited and reticulated on half the surface of 4.8 cm^2^ glass cover slips so that the cell populations were exposed to a comparable amount of epoxy with that employed in encapsulated CCD image sensors. The cover slides with epoxy adhesives were then deposited into 6-well microtiter plates and seeded with RPE1 cells. Some wells of the 6-well microtiter plates were filled only with RPE1 cells to be used as reference conditions for adherence, proliferation and morphology of the cells. The microtiter plates were incubated at 37 °C. Images of the cell populations growing close to the epoxy layers were recorded every 24 h for 3 days using a DP20 phase contrast microscope from Olympus.

### Illumination

Illumination of cell cultures was provided by a white, directional 5 mm Light Emitting Diode (LED) NSPW500BS from Nichia (Tokushima, Japan). The emission spectrum and the radiation pattern of the white LED were characterised using a CCD spectrometer LCS-100 and a goniometric spectroradiometer LCS-100-G from Labsphere (North Sutton, USA), respectively. The LED was placed at about 5 cm above the image sensor without any intermediary optical filter nor pinhole. The LED, the cell culture and the image sensor were enclosed in a black box to prevent the impingement of stray light on the image sensor. The black box was not hermetic so as to enable gas exchange between the culture medium and the cell incubator atmosphere.

### Cell cultures

Adherent human Retinal Pigmented Epithelial cells (RPE1), Human Bronchial Epithelial cells (16HBE), human cervical cancer cells (HeLa) and human Prostate Cancer cells (PC3) were cultured and imaged at 37 °C in a cell culture incubator with a humidified atmosphere containing 5.0% CO_2_. The cell cultures were covered by 1 to 4 mm of medium. 16HBE were grown in MEM enriched with 3 mM L-glutamine, 20 mM Hepes, 10% Fetal Calf Serum (FCS) and 200 μg/mL gentamicin. PC3 were grown in RPMI GlutaMAX supplemented with 10% FCS and 1% penicillin and streptomycin (P/S). HeLa and RPE1 were grown in DMEM/F12 supplemented with 10% FCS and 1% P/S. These standard culture media contained phenol red. The contact images presented in this article were thus captured in usual coloured media. All culture media were supplied by Life Technologies.

### Cell substrates

Borosilicate glass wafers with thicknesses between 50 and 300 μm were purchased from Schott (Mainz, Germany). 500 μm-thick quartz wafers were supplied by Mondia Quartz (Le Versoud, France). 150 μm-thick cover slips and 1 mm-thick microscope slides were supplied by Knittel Gläser (Braunschweig, Germany). These glass slides were diced in samples of a few mm^2^ using the saw cutting machine ProVectus 7100 from Advanced Dicing Technologies (Haifa, Israel). The glass substrates were used individually or stacked to provide various thicknesses, *e.g.*, a 75 μm-thick glass sample was placed on a 500 μm-thick glass sample to achieve a height of 575 μm.

Cell adhesion and growth were favoured by coating the top surface of glass samples with fibronectin. To do so, the glass samples were first cleaned with ethanol 96% for 15 min, dried with argon flow, and exposed to each side with oxygen plasma (30 W, 30 s; Femto system from Diener Electronic, Nagold, Germany) to oxidise both surfaces. The samples were immersed in 0.1 mg/mL poly(L-lysin)-*grafted*-poly(ethylene glycol) PLL(20)-g[3.5]-PEG(2) (PLL-PEG) from SuSoS (Dübendorf, Switzerland) for 30 min to provide an anti-adhesive coating for the cells, rinsed with PBS, rinsed with deionised water and dried. PLL-PEG was removed from the top surface of the glass samples by UV-ozone cleaning with a low pressure mercury lamp NIQ 60/35 XL from Heraeus Noblelight France (Courtaboeuf, France) at a distance of 5 cm (15 mW/cm^2^) for 5 min. The glass samples were then immersed in 10 μg/mL fibronectin (Sigma-Aldrich) for 30 min and rinsed twice with PBS. The achieved double-functionalised cell substrates were ready for cell seeding, with cell adhesion only possible on the fibronectin-coated top side.

### Scanning Electron Microscopy

Dimensions of RPE1 cells were determined using a Scanning Electron Microscope (SEM) ULTRA-55 from Zeiss (Germany). After seeding and growth on a tissue culture-treated substrate, RPE1 cells were fixed, dried and covered by a nanometre-sized Au layer for visualisation by SEM. The images were acquired by the in-lens detector of the SEM, at an accelerating voltage of 15 kV which is an appropriate voltage for biological specimens.

### Ray tracing simulations

Geometrical optics simulations were achieved using optical design software ZEMAX-EE (Zemax, Redmond, USA). Ray tracing simulations were performed on 30 × 30 and 40 × 40 arrays of 1.4 × 1.4-μm pixels which are the smallest pixel size used in this study. As the ray distribution of the background is deformed at the border pixels of the simulated images, 1 or 2 outer pixel lines were removed to produce undistorted images of 28 × 28 pixels (Fig. 2a,b) and 36 × 36 pixels (Fig. 3a,b). Ray tracing was computed with both coherent and incoherent lights. Incident illumination used in the simulations was collimated and orthogonal to the cell substrate. All the incident rays reaching a pixel were assumed to be collected by the underlying photodiode and to contribute to the final image, *i.e.*, possible reflection, scattering and diffraction effects inside the pixel architecture^64^,^65^, spatial optical crosstalk between adjacent pixels^64^ and flawed conversion of incident photons to photoelectrons resulting from limited quantum efficiency were not taken into account.

### Image processing

Non-uniform illumination was corrected in Figs 6a,f, 7a and 8a and Figure S8 using a median filter-generated background image with ImageJ v. 1.41o (NIH, Bethesda, USA). Other images were not modified. Thresholding of cells on the images was performed with ImageJ using a bandpass filter. The circularity index was calculated using the formula 
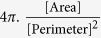
, ranging from 1 for a circle to 0 for an infinitely elongated polygon. The ImageJ plug-in “Manual Tracking” programmed by Fabrice Cordelières (Institut Curie, Orsay, France) was employed to monitor and quantify cell motility.

## Additional Information

**How to cite this article**: Gabriel, M. *et al.* Time-lapse contact microscopy of cell cultures based on non-coherent illumination. *Sci. Rep.* **5**, 14532; doi: 10.1038/srep14532 (2015).

## Supplementary Material

Supplementary Movie S1

Supplementary Movie S2

Supplementary Movie S3

Supplementary Information
