# Supplementary material for: Time-lapse contact microscopy of cell cultures based on non-coherent illumination
Source: Sci Rep. 2015 Oct 13;5:14532. doi: 10.1038/srep14532 (PMC4602279; doi:10.1038/srep14532)
Supplement: Supplementary Information [file srep14532-s4.pdf]

Supplementary Information

Time-lapse contact microscopy of cell cultures based on non-coherent illumination

Marion Gabriel, Dorothée Balle, Stéphanie Bigault, Cyrille Pornin, Stéphane Gétin, François Perraut, Marc R. Block, François Chatelain, Nathalie Picollet-D’hahan, Xavier Gidrol, Vincent Haguet

Supplementary Movies

Movie S1.

The Movie S1 shows real-time sedimentation, rolling and attachment of RPE1 cells onto a 175 µm-thick fibronectin-coated glass substrate positioned on the pixel area of an image sensor. A flow of the cell suspension from the top right corner to the bottom left corner of the image is displayed. The cells in suspension become sharper as they move closer to the image sensor. Cells immobilised on the glass surface appear to have a dark or bright pattern likely depending on their advancement in their adhesion process. Cells sedimenting or rolling on the fibronectin-coated surface are also observed.

Movie S2.

The Movie S2 shows adhesion, spreading and proliferation of RPE1 cells for 33h15 on a 175 µm-thick fibronectin-coated glass substrate positioned on the pixel area of an image sensor. The images were acquired every 4 minutes and are here displayed at 24 fps.

Cells sedimented either individually (dark patterns then becoming bright patterns within the first seconds of the movie) or are deposited in cell aggregates (*e.g.*, an aggregate of cells settles near the bottom left corner at the beginning of the movie, then forming a cluster of cells). After attachment and adhesion, the RPE1 cells spread, move and divide on the surface. Cell mitosis and separating daughter cells can be noticed at this frame rate as brief small bright spots which are particularly perceptible within the clusters of cells. Cell clusters progressively grow, especially a large near-confluent group of cells at the bottom left corner. The 1.1 mm-long alignment of cells observed in the first seconds of the film on the right part of the image may reveal a locally inhomogeneous fibronectin deposition.

The dark circle on the top part of the image is a bubble present on the border of the imaging area. Interestingly, cells can partly move under this bubble. Traces of glob top Vitralit 1690 employed to protect the metal parts of the image sensor are visible at the bottom left and bottom right corners. A transparent triangular trace is also deposited on the pixel array in the bottom left part of the image sensor. Additionally, Brownian motion of a small bubble and of cells in suspension seemingly slipped between the image sensor surface and the glass substrate can be visualised on the bottom part of the movie.

Movie S3.

The Movie S3 shows heat-induced mortality of RPE1 cells for 3 hours on a 175 µm-thick fibronectin-coated glass substrate positioned on the pixel area of an image sensor. This experiment was made immediately after the images displayed in the Movie S2. The images of the Movie S3 were acquired every 4 minutes and are here displayed at 10 fps.

Heating is triggered at the beginning of the movie. Every cell in the culture contracts and rounds up as the temperature rises up to 52 °C. A growing bubble on the top border of the imaging area pushes the nearby cells. The large and dynamic shadows recorded in the movie are generated by the condensation and motion of droplets on the lid of the Petri dish as a result of heating-induced evaporation of water. The cell aggregates slipped between the image sensor surface and the glass substrate, at the bottom of the image, do not move under overheating exposure, which suggests that those cells are also engaged toward mortality.

Supplementary Figures

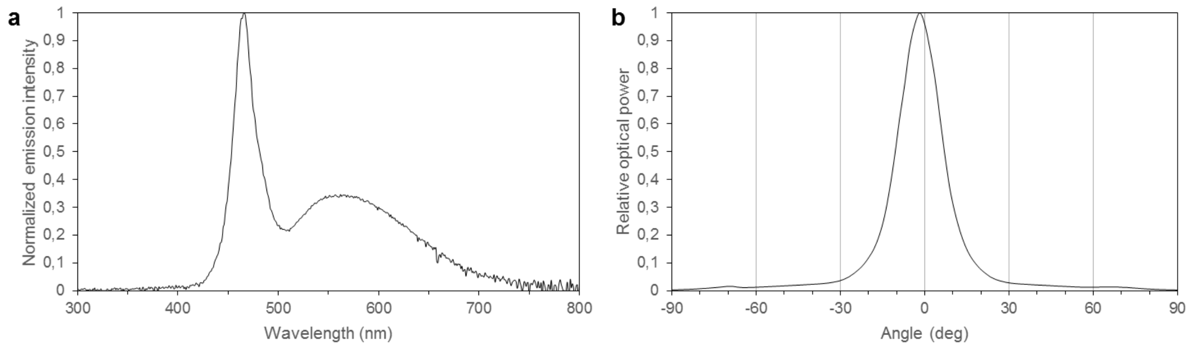

Figure S1 Radiation characteristics of the light source with a nominal current of 30 mA. a) Emission spectrum of the employed LED measured by spectrometry. b) Radiation pattern of the LED characterised by goniometric spectroradiometry. The slight directivity shift of the LED toward the -1.8° angle, indicative of a non-optimal packaging process typically implemented to manufacture cheap LEDs, can be considered to be negligible for the aim of our work.

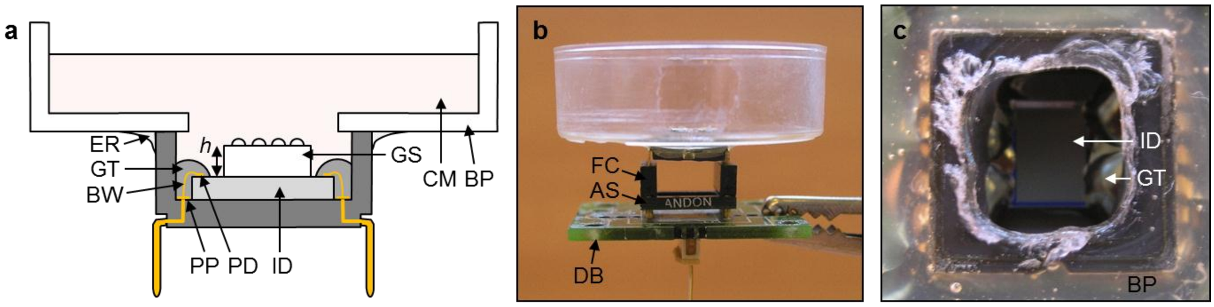

Figure S2 Cell imaging device. a) Side view diagram of the experimental setup. After removal of the glass lid covering the image sensor, some glob top was deposited on either side of the silicon imaging area to insulate the metal connections from cell culture medium. A culture chamber was made of a pierced 35 mm Petri dish glued on the image sensor package. b) Side view of the image sensor fixed on female connectors and an Andon socket on the daughterboard. c) Top view of the image sensor with the hole in the bottom of the Petri dish. ID: image sensor die. GS: glass substrate of thickness *h* with cells on the top surface. CM: cell medium. BP: bottom-pierced Petri dish. PP: pad of the plastic package. PD: pad of the image sensor die. BW: bonding wire. GT: glob top. ER: epoxy resist. FC: female connector. AS: Andon socket. DB: daughterboard.

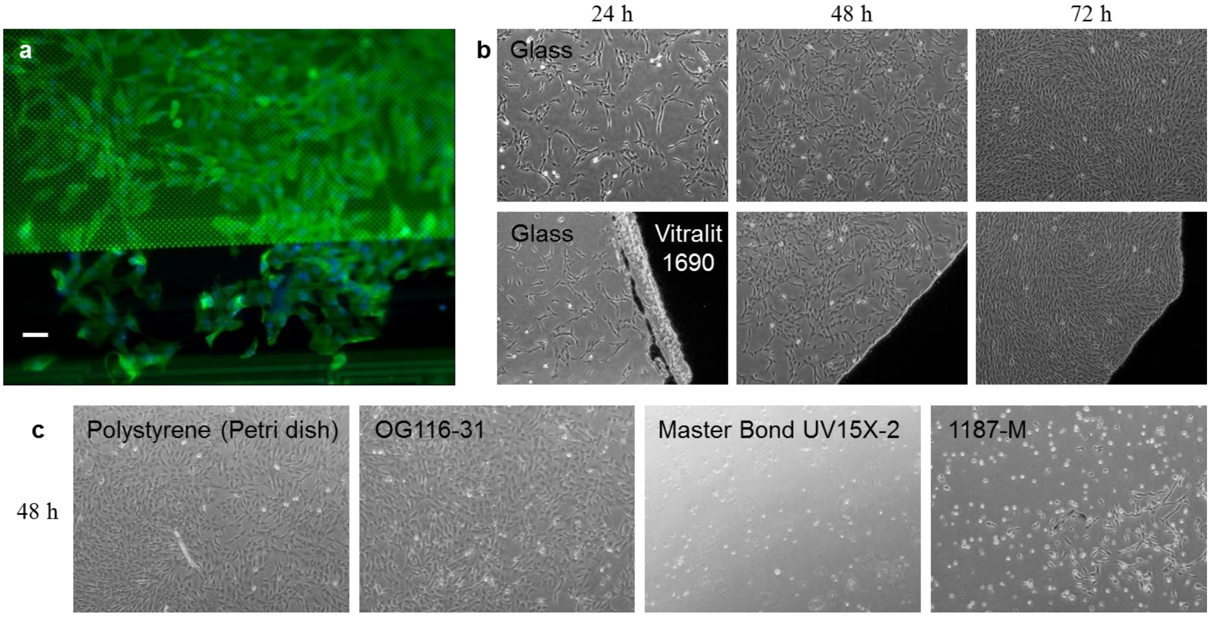

Figure S3 Biocompatibility assays of the cell imaging device. a) Fluorescent microscopy image of RPE1 cells after 16 h of culture on a colour image sensor surface, fixation, and Hoechst and phalloidin-FITC labelling of the nucleus (blue) and actin (green), respectively. RPE1 cells have adhered on the image sensor and spread with normal morphology. The green pixels of the image sensor are also apparent on the fluorescence image. Scale bar: 50 µm. b) Proliferation and morphology of RPE1 cells in the presence of Vitralit 1690 (Eleco Produits, Gennevilliers, France) are very similar to that on the glass substrate. c) RPE1 cells in the presence of OG116-31 (Epoxy Technology, Billerica, USA) present normal adherence, proliferation and morphology. On the opposite, Master Bond UV15X-2 (Master Bond, Hackensack, USA) and 1187-M (Dymax, Torrington, USA) certainly release chemicals altering cell metabolism, viability or proliferation rate. Other UV curable epoxy adhesives tested negatively are 1160-M, 1165-M, 1180-M and 203A-CTH-F from Dymax.

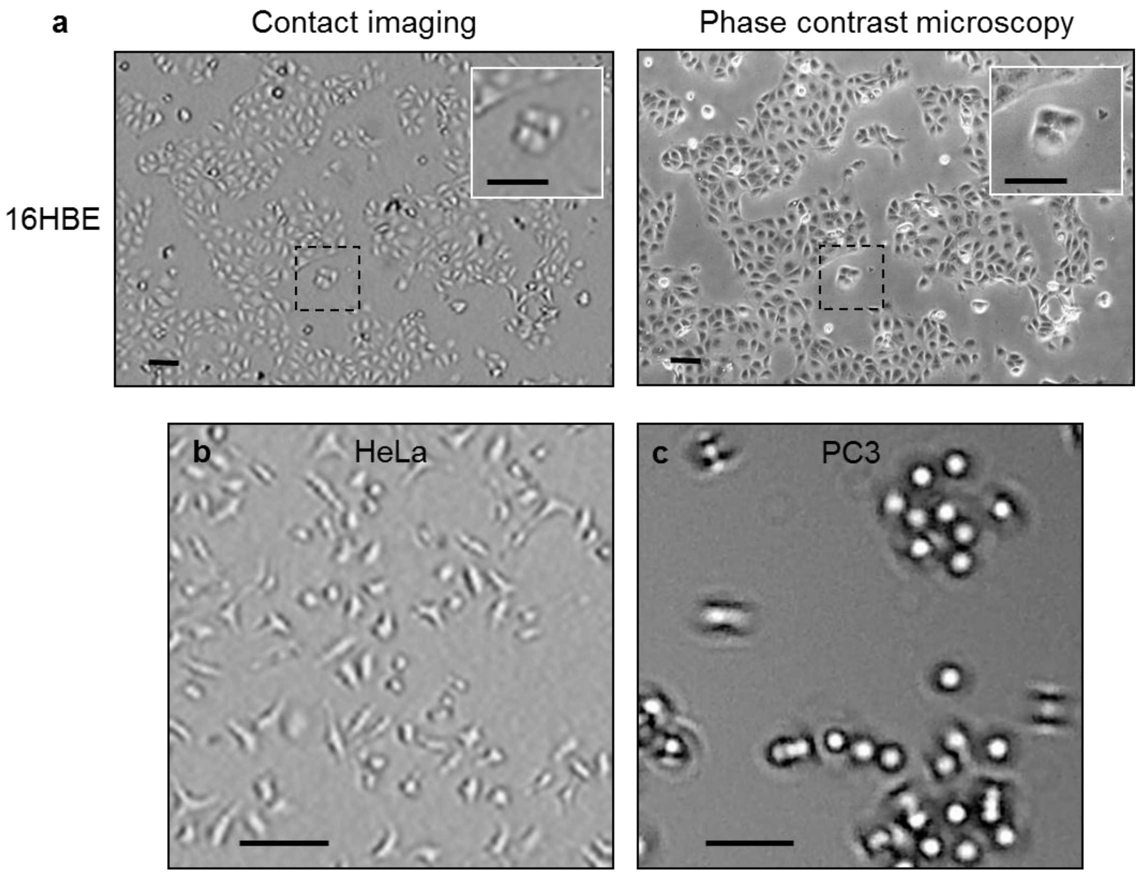

Figure S4 Images of 3 human cell lines. a) 16HBE cells adhered on a fibronectin-coated glass slide (h=175 µm) visualised by contact imaging with a CCD image sensor ICX098 and by phase contrast microscopy (Olympus DP20, magnification 4×). b,c) Contact images of HeLa cells (h=175 µm) and PC3 cells (h=575 µm) visualised using a 5.6-µm-pixel CCD image sensor and a 1.4-µm-pixel CMOS image sensor, respectively. Scale bars: 100 µm.

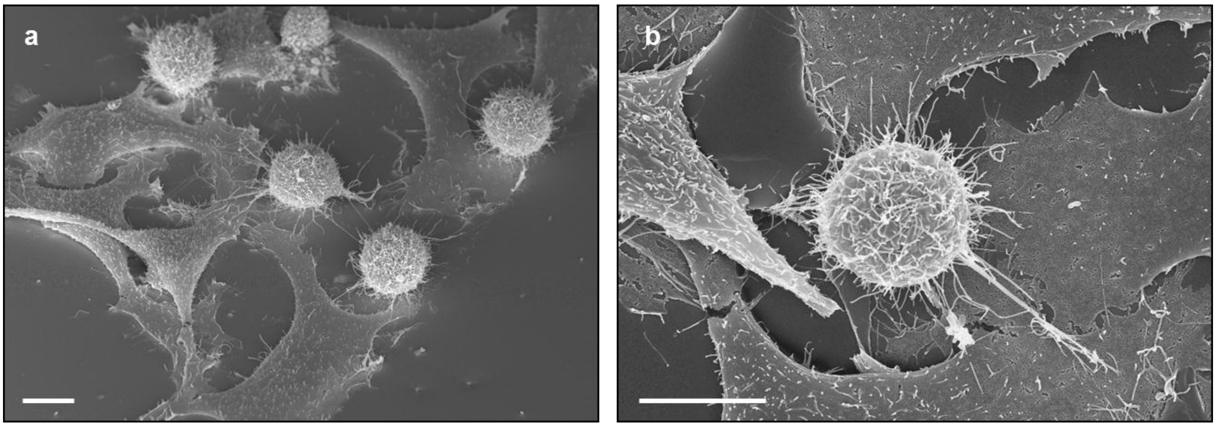

Figure S5 Scanning Electron Microscopy images of RPE1 cells fixed during mitosis (round cells) and interphase (flat cells). SEM images were used to determine the cell dimensions for the ray tracing simulations in Figure 2a,b and Figure 3a,b in the main text. Filopodia tethering the cells to the fibronectin-coated substrate are visible on the cell surface. Scale bars: 10 µm. Image courtesy of Manuel Théry, CEA Grenoble.

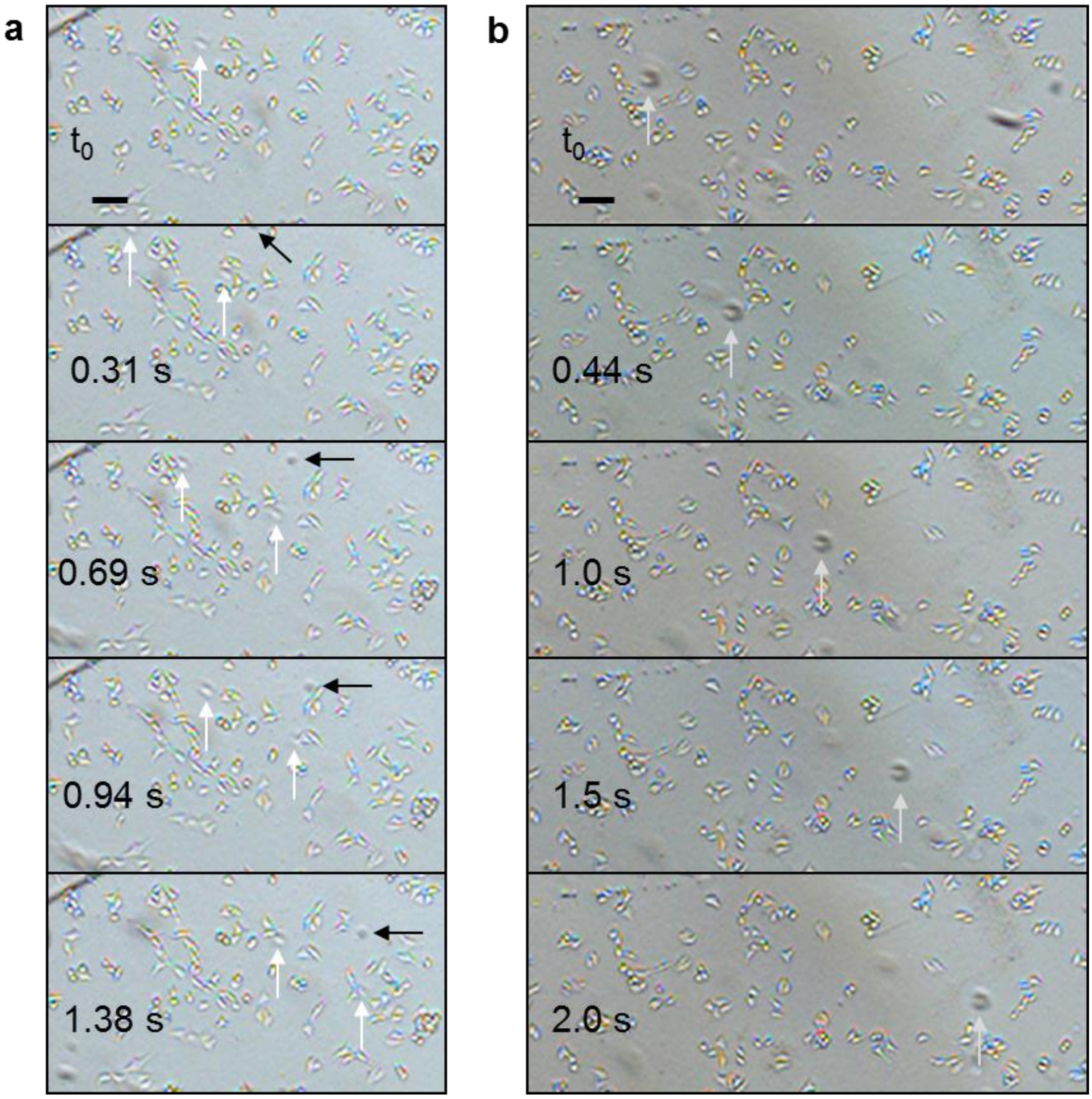

Figure S6 HeLa cells in suspension flowing at short distances from the image sensor. The image sensor records the photonic nanojet emerging from spherical cells [two white arrowed cells in (a)], the end of the photonic nanojet [arrowed cell in (b)] or a dark pattern [black arrowed cell in (a)], depending on the distance of the flowing cells to the image sensor. Non-moving HeLa cells present in the images are adhered on a 50 µm-thick fibronectin-treated glass slide. Scale bars: 100 µm.

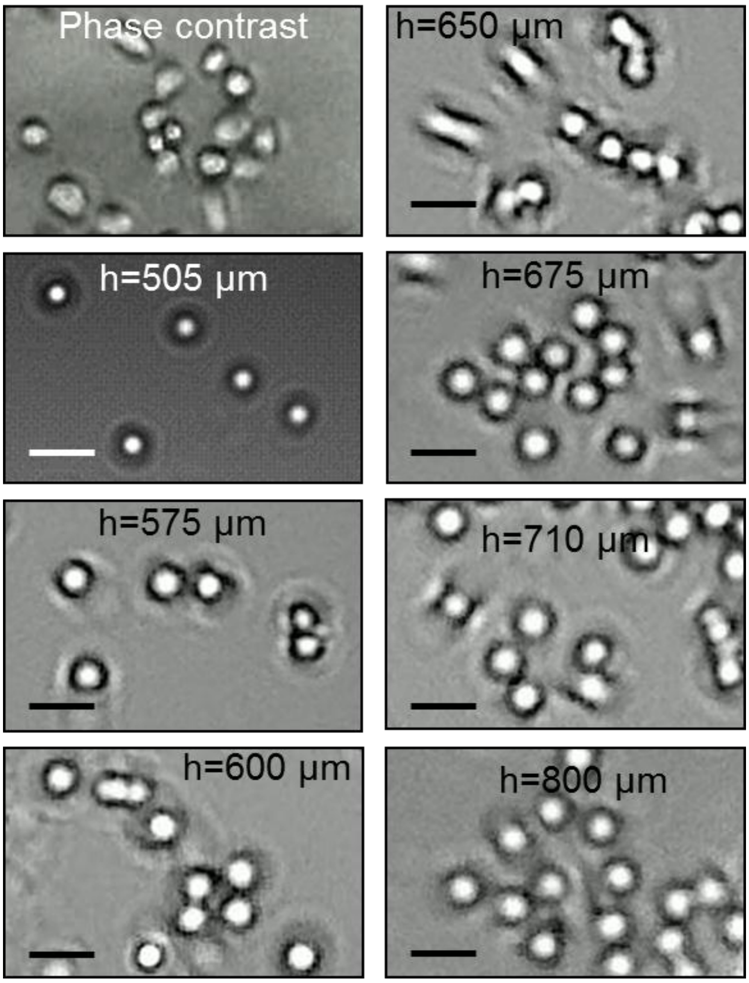

Figure S7 (Top left image) Adherent PC3 cells in interphase observed in phase contrast microscopy at magnification 4×. PC3 cells retain a round shape even in interphase and can thus be used as a model for round adhered cells. Image courtesy of Sophie Gerbaud, CEA Grenoble. (Seven other images) Unprocessed contact images of adhered PC3 cells in interphase acquired at various heights *h* between 505 µm and 800 µm under incoherent illumination. Scale bars: 50 µm.

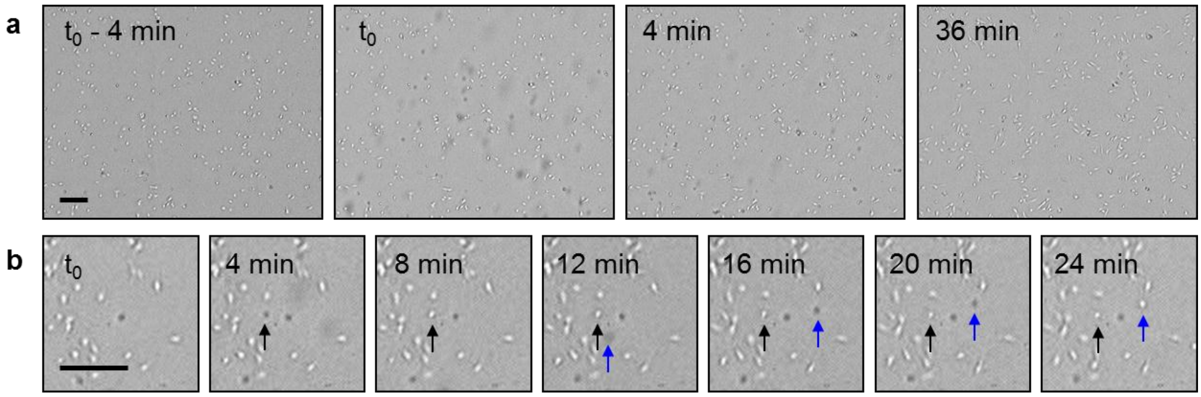

Figure S8 Detachment of cells by mechanical vibrations produced by the incubation door. a,b) Some cells deposited on a fibronectin-coated glass slide (h=175 µm) returned back in suspension when the door of the incubator was gently opened and closed 40 minutes after cell seeding. Most of the detached cells sedimented again within 10 min. Some remained in suspension until ~35 min. This observation reports that, while being common events in a cell biology laboratory, opening and closing the incubator door have an impact on insufficiently adhered cells, even a few tens of minutes after seeding onto a surface coated with cell adhesion molecules. b) Redeposition and adherence of two cells (arrows). Adherence of the cells is noticed by a change of their appearance from dark patterns to bright patterns with a dark contour. Two dark cells, deposited on the surface but likely having not completed their adherence process, are also visible on the image sequence. Scale bars: 200 µm.
